# Equivalent susceptibility of *Anopheles gambiae* M and S molecular forms and *Anopheles arabiensis* to *Plasmodium falciparum* infection in Burkina Faso

**DOI:** 10.1186/1475-2875-12-204

**Published:** 2013-06-14

**Authors:** Awa Gnémé, Wamdaogo M Guelbéogo, Michelle M Riehle, Antoine Sanou, Alphonse Traoré, Soumanaba Zongo, Karin Eiglmeier, Gustave B Kabré, N’Falé Sagnon, Kenneth D Vernick

**Affiliations:** 1 Centre National de Recherche et de Formation sur le Paludisme, Ouagadougou, Burkina Faso; 2 Université de Ouagadougou, Ouagadougou, Burkina Faso; 3 Department of Parasitology and Mycology, Unit of Genetics and Genomics of Insect Vectors, Institut Pasteur, Paris, France; 4 CNRS Unit of Hosts, Vectors and Pathogens (URA3012), Paris, France; 5 Department of Microbiology, University of Minnesota, Minneapolis, Minnesota, USA

**Keywords:** *Anopheles gambiae*, *Anopheles arabiensis*, *Plasmodium falciparum*, Malaria vector, Genetic susceptibility, Molecular form, Infection prevalence, Infection intensity

## Abstract

**Background:**

The *Anopheles gambiae* sensu lato (s.l.) species complex in Burkina Faso consists of *Anopheles arabiensis,* and molecular forms M and S of *Anopheles gambiae* sensu stricto (s.s.). Previous studies comparing the M and S forms for level of infection with *Plasmodium falciparum* have yielded conflicting results.

**Methods:**

Mosquito larvae were sampled from natural pools, reared to adulthood under controlled conditions, and challenged with natural *P. falciparum* by experimental feeding with blood from gametocyte carriers. Oocyst infection prevalence and intensity was determined one week after infection. DNA from carcasses was genotyped to identify species and molecular form.

**Results:**

In total, 7,400 adult mosquitoes grown from wild-caught larvae were challenged with gametocytes in 29 experimental infections spanning four transmission seasons. The overall infection prevalence averaged 40.7% for *A. gambiae* M form, 41.4% for *A. gambiae* S form, and 40.1% for *A. arabiensis*. There was no significant difference in infection prevalence or intensity between the three population groups. Notably, infection experiments in which the population groups were challenged in parallel on the same infective blood displayed less infection difference between population groups, while infections with less balanced composition of population groups had lower statistical power and displayed apparent differences that fluctuated more often from the null average.

**Conclusion:**

The study clearly establishes that, at the study site in Burkina Faso, there is no difference in genetic susceptibility to *P. falciparum* infection between three sympatric population groups of the *A. gambiae* s.l. complex. Feeding the mosquito groups on the same infective blood meal greatly increases statistical power. Conversely, comparison of the different mosquito groups between, rather than within, infections yields larger apparent difference between mosquito groups, resulting from lower statistical power and greater noise, and could lead to false-positive results. In making infection comparisons between population groups, it is more accurate to compare the different groups after feeding simultaneously upon the same infective blood.

## Background

Malaria remains a major global health problem despite widespread control efforts. Many current malaria control measures use interventions aimed at limiting human-vector contact. Insecticide-treated bed nets (ITNs) coupled with indoor residual spraying (IRS) are the principal interventions employed across Africa. However, increasingly widespread insecticide resistance encourages the implementation of integrated or novel vector control methods [1]. In addition, the complexity of vector populations further complicates design and implementation of any control strategies, novel or existing, and argues against a one-size-fits-all control strategy. The *Anopheles gambiae* species complex is heterogeneous, comprised of seven morphologically indistinguishable species that vary in their distribution, ecology and contribution to malaria transmission. *Anopheles gambiae* sensu stricto (s.s.) and *Anopheles arabiensis* have the most prominent roles in malaria epidemiology, with *A. gambiae* receiving the most research attention due to its high degree of anthropophily and reference genome sequence. However, concentrating solely on reducing malaria transmission by *A. gambiae* without consideration of other potential vectors may not yield long-term control of malaria transmission [2,3].

Genetic subdivision within *A. gambiae* allows fine ecological partitioning of the species, resulting in spatial and temporal expansion of malaria transmission [4,5]. Two population subgroups of *A. gambiae*, referred to as molecular forms, serve as an example. The S form exists across the entire range of *A. gambiae* s.s., while a second subgroup, the M form, is found only in West and Central Africa. The M form may represent an example of niche expansion by a founder population, because it dominates in marginal and disturbed habitats where the S form is comparatively less competitive [6-8]. Further, the M form shows a greater ability to exploit more permanent breeding sites, including sites created by irrigation, rice cultivation and urbanization [6,9-11]. This adaptation may allow the M form to breed throughout the year in some locations, thus potentially causing a shift from seasonal to year-round malaria transmission [12].

The molecular forms display pronounced genetic differentiation near the centromeric regions of each chromosome, accompanied by other heterogeneous differentiation throughout the genome [13-15]. The canonical M and S forms are reproductively isolated at the prezygotic stage [16], which gave rise to the hypothesis that they may be incipient species. However, more recent work has described some populations with high rates of apparent hybridization and introgression between molecular forms [12,17,18]. An outdoor-resting subgroup of *A. gambiae* s.s., called Goundry, is genetically distinct from both the M and S forms, and the M and S diagnostic markers segregate freely in complete equilibrium [19]. These disparate observations will probably only be resolved when large-scale sequencing of individual mosquitoes allows fine-grained genetic and geographic analyses.

The molecular forms show signs of a selective sweep at two immune gene loci in the M form, but not in S form [20,21]. In a survey of other immune genes, each population group displayed distinct signatures of immune selection in different genes, and no overlap of gene signatures was seen across population groups [22]. This observation is consistent with the idea that the population groups are exposed to different pathogen selective pressures [20,21].

Population genetic differences between subgroups might cause differential levels of infection between the subgroups. There are two main ways to test for potential differences between subgroups: measuring genetic susceptibility, or determining natural infection rate. Here, genetic susceptibility is compared between *A. gambiae* forms M, S, and *A. arabiensis*. “Genetic susceptibility,” also called inherent susceptibility, measures the genetically-controlled physiological permissiveness for parasite development in mosquitoes under conditions that eliminate or control for as many environmental variables as possible, in order to detect only genetically-based differences between subgroups. This is done using mosquitoes that are raised and exposed to infective blood under conditions that control mosquito age, previous infection, environmental history, and other factors. Alternately, “natural infection rate” is based on infection prevalence rates of wild-captured adult mosquitoes, where the probability of infection summarizes all environmental and genetic variables, including host choice, temperature and humidity, age, survival rate, in addition to any genetic differences between subgroups.

Several studies have compared natural infection rates between M and S forms in wild-captured adult mosquitoes, and none of them detected any difference in infection rate between M and S forms [23-25]. In the two previous studies of genetic susceptibility, one in Senegal reported higher prevalence and intensity of infection in S form as compared to M [26], while a study in Cameroon reported higher infection prevalence in the opposite direction [27].

The current study examines the genetic susceptibility of wild *A. gambiae* sensu lato (s.l.) mosquitoes in Burkina Faso to infection by wild genotypes of *P. falciparum*. A large number of mosquitoes and parasite genotypes were tested over four years. Although there is fluctuation between individual infection experiments, there is no overall effect of species or molecular form upon the level of mosquito infection. Interestingly, infection experiments in which the population groups were challenged in parallel on the same infective blood displayed less infection difference between population groups, while infections with less balanced composition had lower statistical power and departed more often from the null average. This analysis indicates that in making infection comparisons between population groups, it is more accurate to compare the different groups after feeding simultaneously upon the same infective blood.

## Methods

### Study site

The study used wild populations of *A. gambiae* s.l. and natural gametocytes of *P. falciparum*. Data were collected during four consecutive years, from 2007 to 2010, in the rainy season (August-November). Mosquitoes were collected as stage 3 and 4 larvae at the site of Goundry (12°30' N and 1°30' W), a rural village in the Sudan-Savanna ecological zone located 35 km north-east of Ouagadougou in Burkina Faso. The landscape is modified by human activity, creating many mosquito breeding sites during the rainy season, including a dam, mud brick pits, small ponds, hoof prints, puddles, and streams. Four population groups of *A. gambiae* s.l. are sympatric in and around Goundry, the *A. gambiae* s.s. groups M, S, and Goundry, and sibling species *A. arabiensis*[19,28]. The susceptibility of the Goundry subgroup to *P. falciparum* infection was reported elsewhere [19], and as its role as a natural vector is not yet known, samples of the Goundry subgroup were not analysed as part of the current study.

Gametocyte carriers were identified in Laye, a village situated 30 km north-west of Ouagadougou, about 60 km from Goundry. As with Goundry, Laye is situated in the Sudan-Savanna zone with one rainy season from August to November. Laye was used because it is part of the Demographic Surveillance System (DSS) used by the Centre National de Recherche et de Formation sur le Paludisme (CNRFP), the national malaria research centre for epidemiological studies. Epidemiological characteristics of the study site were recently described [29]. There is little geographic population structure of *P. falciparum* in Africa [30] and populations from Burkina Faso and Mali were not genetically distinct [31], so the small distance between Laye and Goundry should have no effect.

### Mosquito collection

Throughout the rainy season ~50 different breeding sites were visited twice a week and mosquito larvae were collected by the standard ladle dipping method. Ten larvae from each of the 50 larval sites were pooled to create an experimental cohort of ~500 individuals. The larval contribution per breeding site was limited in order to avoid potential bias from oversampling siblings. The larvae from a collection cycle were reared in the CNRFP insectary in the water taken from their larval pool, after filtering to remove predators and debris. As water evaporated, the larval pans were topped up with distilled water. Otherwise, standard larval rearing procedures were used. In Goundry, larval sites are shared by M and S forms and *A. arabiensis*, and significant partitioning of molecular forms by pool has not been detected (of 66 genotyped single-pool larval collections, 11 were comprised of M form only, seven were *A. arabiensis* only, which reflects the greater overall prevalence of M form and *A. arabiensis*, and 48 were mixtures of any two or all three groups). Thus, the collection and rearing procedures randomize and therefore control for any hypothetical effects due to differential microbial flora of larval pools, which is unlikely here in any case due to predominant larval site sharing. Emerging adults were fed on 5% glucose, and adults emerging on days 2–4 were pooled in an age-matched group for blood feeding. An age spread this small does not influence infection susceptibility [32].

### Selection and screening of gametocyte carriers

In each experimental round, a group of ten potential gametocyte carriers five to ten years old were screened for malaria parasites by standard finger-prick and Giemsa-stained blood smear. The parasite density was determined for 100 microscopic fields of the blood smear. Assuming a microscope field contains ~20 leukocytes, parasites were counted for 2,000 leukocytes and parasite density was determined for the standard number of 8,000 leukocytes/μl of blood. The screening took place the same day as the experimental infection of mosquitoes. Carriers were chosen based on two criteria, as described and used previously [29]: i) infection with only a single species of malaria parasite, *P. falciparum,* and ii) presence of mature gametocytes. For carriers meeting these criteria, 5 ml of venous blood was drawn into heparinized tubes for experimental feedings.

### Experimental infections

For the infection experiments, female mosquitoes were starved for 12–15 hours prior to the infectious blood meal. Freshly drawn blood was immediately transferred to artificial membrane feeders pre-warmed to 37°C as described previously [33,34]. Mosquitoes were allowed to feed for 15 min, after which unfed mosquitoes were removed. Fed mosquitoes were maintained in the insectary on 5% glucose solution. Physical removal of unfed mosquitoes is highly efficient, but blood meal was confirmed for subsamples of mosquitoes by determining Christopher’s stages of ovarian development at the time of subsequent dissection [35]. The vast majority of unfed mosquitoes (91%, n=150) were *A. arabiensis*. A genetic analysis comparing fed and unfed *A. arabiensis* using eight microsatellites on chromosome 3, analysed using Genepop as previously described [19], showed no detectable genetic differentiation between fed and unfed *A. arabiensis* (Fst=0). Thus, the unfed *A. arabiensis* are simply a random subset of the total, and the results of genetic susceptibility to *P. falciparum* are valid for *A. arabiensis* as a whole. The presence of oocysts (infection prevalence), and oocyst number for mosquitoes carrying at least one oocyst (infection intensity) was determined seven to eight days post-blood meal by dissection of midguts, staining with 2% mercurochrome, and light microscopy. Carcasses were stored for later DNA extraction.

A quality control (QC) filter was imposed to select the most informative infections. These were defined as infection sessions with overall infection prevalence ≥30% and maximum intensity in at least one individual mosquito of ≥10 oocysts. Note that the QC filter was imposed on the entire infection session, not on individual mosquito samples. Twenty-nine infection sessions satisfied the QC, and all of the mosquitoes from these 29 sessions were used in further analysis, while the mosquitoes from infections that did not satisfy the QC criteria were not used in the analysis. Infections not meeting QC can result from technical variables or biological effects, but since it is not possible to distinguish the cause, infections below QC criteria must be assumed to be enriched for false-negative infections, and were considered to be unreliable and mostly a source of noise. The same quality cut-off has been used previously for field infections [19,34,36]. A sample of infections not meeting QC were genotyped, and the population group composition was indistinguishable from the infections that did satisfy QC (*A. gambiae* M form p=0.551, *A. gambiae* S form p=0.175 and *A. arabiensis* p=0.625). There is also no significant difference between the overall sample size of infections that meet the QC threshold and those that do not (p=.957). Thus, the infection QC filter reduces noise, increases data quality, and does not introduce a genetic bias.

### Genotype analysis

Genomic DNA was extracted from mosquitoes using DNAzol by the supplied protocol (Invitrogen, CA, USA) and samples were genotyped as described previously [19]. Briefly, species and molecular form were typed by either or both of two widely used molecular diagnostic assays [37,38]. As described in detail elsewhere, assignment to either the M, S, or Goundry subgroups was done by Bayesian clustering methods employed by STRUCTURE, analysing microsatellite genotype data from eight microsatellites on chromosome 3 [19,39], which assigned >95% of mosquitoes to a subgroup at >80% probability. The current study analysed the canonical reproductively isolated *A. gambiae* s.s. M and S forms (the Endo group in [19]) as well as sister taxon *A. arabiensis*.

### Statistical analysis

Infection prevalence was compared between three population groups (*A. gambiae* M form, S form, and *A. arabiensis*) using the Wilcoxon Signed Rank Test. The complete sample set analysed was comprised of 29 infections that met the QC criteria described above (n=2,311 mosquitoes). In each of these 29 infections, all three of the population groups were represented by ≥1 mosquito each. A subset of 13 of these 29 infections, in which all three population groups (*A. gambiae* M form, S form, and *A. arabiensis*) were represented within the same infection by at least seven mosquitoes each were subject to additional analysis. This subset of 13 most representative infections has greater statistical power because between-infection variables were controlled, including human host factors, parasite genotype, seasonal fluctuation, and technical variation. In addition to the Wilcoxon test, the 13 infections were also analysed by chi-square test followed by the combination of *p*-values across all infections for a given pair-wise comparison via the Fisher method [40]. The variance of the difference in infection prevalence between M and S forms was calculated by taking the absolute value of the difference in infection prevalence between M and S form within each of the 29 infections, and then calculating the variance of these differences. Infection intensity between population groups was compared using the Wilcoxon Signed Rank Test. Regression analyses were done to examine the correlation between infection prevalence and infection intensity. These analyses were done within a population group for the 13 most representative infections (as described above). For all analyses described, p<0.05 was the threshold for statistical significance.

### Ethical considerations

The study protocol was reviewed and approved by the institutional and national health ethical review board (Commission Nationale d’Ethique en Santé) of Burkina Faso (code N° 2006–032). The study procedures, benefits and risks were explained to parents or legal guardians of children and their informed consent was obtained. Children of parents or guardians who had given consent were brought to CNRFP the day of the experiment for gametocyte carriers screening. All children were followed and symptomatic subjects were treated with the combination of artemether-lumefantrine (Coartem®) according to relevant regulations of the Burkina Faso Ministry of Health.

## Results

### Experimental infections

During the four-year study period, a total of 830 children five to ten years old were screened for *P. falciparum* infection. The prevalence of all malaria parasites was 70.7% in the survey population and 68.20% were *P. falciparum* carriers. Across these infected children, 33.4% presented with *P. falciparum* gametocytes. A total of 110 carriers were used for infection experiments, which yielded 29 infection sessions meeting quality control criteria (see Methods). For these 29 infection sessions, the mean age of gametocyte carriers was 7.6 years and the average gametocyte density was 170.6/μl. Descriptive statistics for the 29 infection sessions are given in Table 1.

**Table 1:** Descriptive statistics for experimental infections

	**Gametocyte carrier characteristics**			**Mosquito characteristics**					
**Statistics**	**Age**^**a**^	**Tf**^**b**^	**Gf**^**c**^	**Exposed**^**d**^	**Fed**^**e**^	**Dissected**^**f**^	**Dead**^**g**^	**Infected**^**h**^	**Mean oocyst number**^**i**^
Minimum	5.3	0	16	150	33	30	0	11	1.541
25% Percentile	6.05	703	73.75	200	65	54.5	3	20	2.503
Median	7.4	1,203	104	230	87	82	7	37	4.9
75% Percentile	9.15	7,473	211	300	104	91.5	11.5	57	9.389
Maximum	12.5	1,8681	1143	450	200	192	26	88	31.2
Mean	7.627	4,056	170.6	255.2	87.52	79.69	7.828	38.59	7.109
Standard Error	0.3356	993.8	38.82	15.52	6.524	6.197	1.254	3.873	1.273
Sum				7,400	2,538	2,311	227	1,119	

All statistics were calculated using data from 29 individual experimental infections.

^a^ Child age in years, ^b^ Trophozoite density per ml of blood, ^c^ Gametocyte density per ml of blood, ^d^ Number of mosquitoes offered a blood meal, ^e^ Number of mosquitoes that took a blood meal, ^f^ Number of mosquitoes dissected seven to eight days following infection, ^g^ Number of mosquitoes that died between feeding and dissection, ^h^ Number of mosquitoes with at least one midgut oocyst, ^i^oocyst intensity (average number of midgut oocysts in mosquitoes with ≥1 midgut oocyst).

In total, 7,400 mosquitoes were offered an infective blood meal via membrane feeding. Of these, 31.0% (2,311/7,400) successfully fed and also survived until dissection one week later for determination of infection prevalence and intensity. After genotyping for species and assignment to mosquito population group, 2,168 mosquitoes from the 29 infections were analysed for comparative susceptibility to parasite infection. The analysed samples by year were n=595, 685 and 395 and 493 for the transmission seasons of 2007, 2008, 2009, and 2010, respectively. Across all study years, the population composition was 36.0% (n=780) *A. arabiensis*, 12.8% (n=277) *A. gambiae* M form, 18.7% (n=406) *A. gambiae* S form and 32.5% (n=705) *A. gambiae* Goundry subgroup. Infection susceptibility of the Goundry group was previously analysed [19], and is not treated further in the current study.

### Infection prevalence among population groups

**Figure 1:**
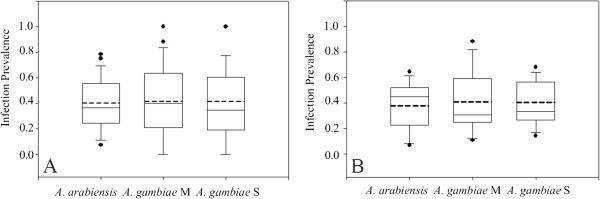
**Genetic susceptibility to *Plasmodium falciparum *infection is the same across *Anopheles gambiae *s.l. population groups. A**. Infection prevalence of the three population groups, *A. gambiae* s.s. M form, S form and *A. arabiensis*, was calculated individually for each of 29 experimental infections, and descriptive statistics are summarized in a box plot (top and bottom of the box, 75th and 25th percentile; solid line within the box, median; dashed line within the box, mean; error bars, 90th and tenth percentiles; dots, outliers). **B**. As in **A**, but only for the subset of 13 most representative infections, with the greatest power for comparison of susceptibility to infection across population groups. Neither the 29-infection dataset in **A** nor the 13-infection subset in **B** display a significant difference for infection prevalence among population groups (see Results).

Mosquitoes fed on different infective blood meals are known to display wide variation in infection distribution due to numerous variables [41-43]. Thus, it is not statistically valid to simply pool the data across all infections and calculate a single prevalence value per population group. Instead, it is necessary to compute the infection prevalence of each population group for each independent infection, and then combine and analyse these infection prevalence values among population groups. The infection prevalence for each population group was determined for each of 29 infections, and then these 29 prevalence values were compared between all population groups. The average infection prevalence across the 29 infections was, *A. arabiensis* (40.1%, n=780), *A. gambiae* M form (40.7%, n=277) and *A. gambiae* S form (41.4%, n=406). Infection prevalence was not significantly different between any of the three groups (Figure 1A, Wilcoxon Signed Rank Test on 29 prevalence values per population group; *A. arabiensis vs A. gambiae* M form *p*=0.897, *A. gambiae* M form *vs A. gambiae* S form *p*=0.779, *A. arabiensis vs A. gambiae* S form *p*=0.690).

The result is unchanged if only the 13 most representative infections are considered. These 13 infections, which consequently have the least noise and the greatest power to detect statistical difference, display equivalent infection prevalence values among population groups: *A. arabiensis* (38.6%, n=356), *A. gambiae* M form (40.2%, n=179) and *A. gambiae* S form (40.2%, n=234) (Table 1 and Figure 1B, Wilcoxon Signed Rank Test on 13 prevalence values per population group *A. arabiensis vs A. gambiae* M molecular form *p*=0.635, *A. gambiae* M form *vs A. gambiae* S form, *p*=1.000 and *A. arabiensis vs A. gambiae* S form *p*=0.519). An alternate method of statistical analysis of the 13 infections, by comparison of individual infection rates using the chi-square test and combining of *p* values using the approach of Fisher, also firmly rejected the hypothesis of infection prevalence differences between any of the population groups (*A. arabiensis vs A. gambiae* M molecular form, *p*=0.996, *A. gambiae* M form *vs A. gambiae* S form, *p*=0.894, *A. arabiensis vs A. gambiae* S molecular form, *p*=0.920).

**Figure 2:**
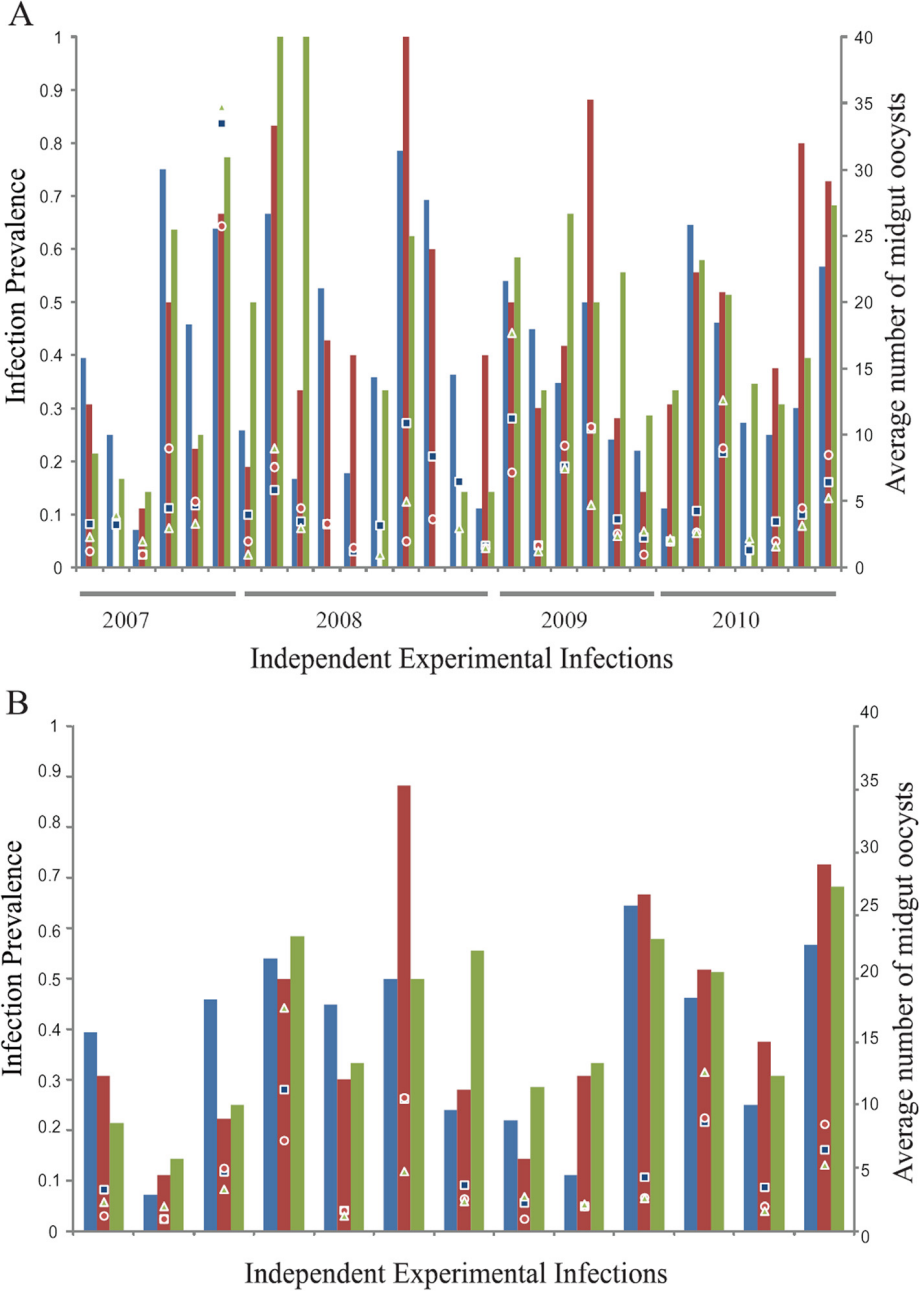
**Infections display wide variation but no tendency of difference between *Anopheles gambiae *s.l. population subgroups.** Values for *Plasmodium falciparum* infection prevalence (bars, primary y-axis) and oocyst intensity (points, secondary y-axis) were calculated for: **A**. all 29 experimental infections and **B**. only the subset of 13 most representative infections. In both **A** and **B**, data for *A. arabiensis* are shown as blue bars and blue squares, data for *A. gambiae* s.s. M form as red bars and red circles and S form as green bars and green triangles. Note that for seven infections shown in 2A, one of the population subgroups has an infection prevalence of 0%, but all population subgroups are represented by ≥1 mosquito in all 29 infections. Population groups within an infection display indistinguishable values for oocyst prevalence and intensity (see Results), and visual inspection of the bars indicates the absence of even a tendency of difference across the population groups. Examination of the 13 most representative infections indicates that, consistent with the statistical analysis (see Results), there is less apparent variation among population subgroups when they are compared within the same infection.

**Figure 3:**
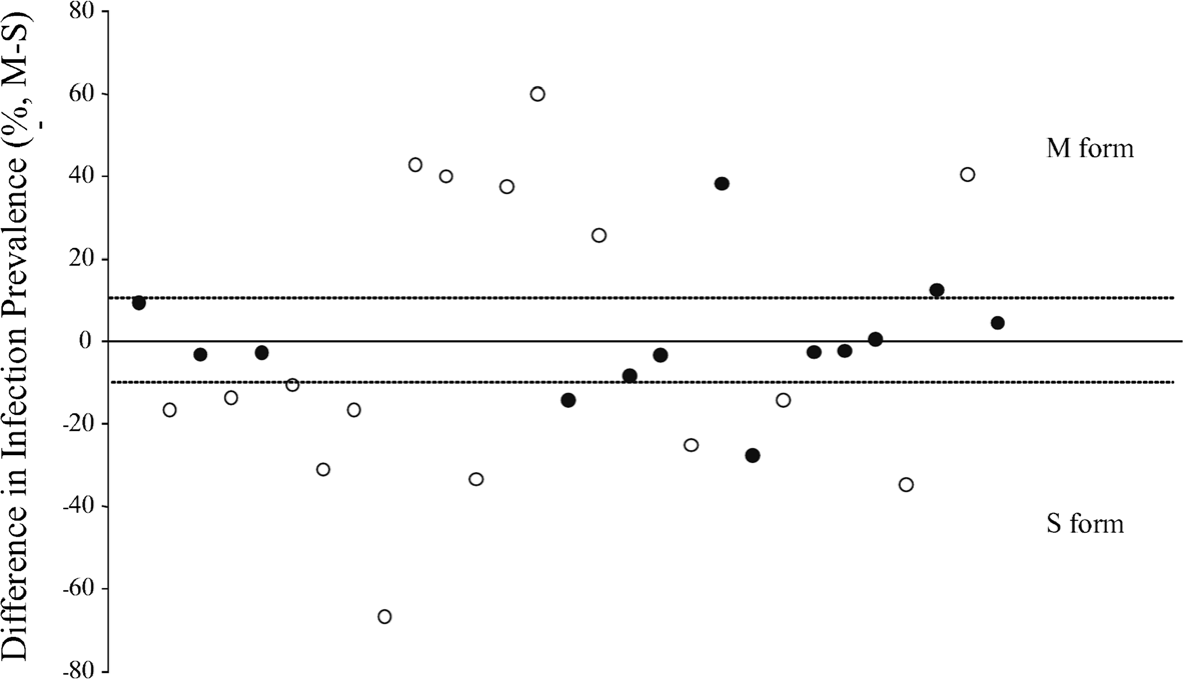
**Difference of infection prevalence between *Anopheles gambiae *M and S forms reveals greater accuracy for comparisons made within an infection.** Each point is the ratio of infection prevalence between *A. gambiae* M and S form for a single experimental infection (n=29 infections). The y-axis is the difference in infection prevalence between molecular forms M and S fed on the same infectious blood meal, and the horizontal line at y=0 is the null average, where infection prevalence of the M and S forms are identical. Positive values indicate infections with greater infection prevalence in M form, and negative values indicate greater S-form infection prevalence. Infections are arranged temporally along the x-axis. Over the 29 infections, there is no significant difference between M and S form infection prevalence (see Results), despite the large variation observed between different infections. Closer examination shows that the subset of 13 most representative infections (closed circles) display less variation across infections, and are clustered closer to the null average than the remainder of infections (open circles) that have unbalanced representation of population groups within the same infection. Thus, 11/13 (85%) of the most representative infections are within 10% of the null average, while only 1/16 (6%) of the remaining less-representative infections are within 10% of the null average. This result strengthens the statistical absence of difference between M and S forms, and indicates that extraneous experimental noise is decreased when comparisons of mosquitoes within the same infection are maximized.

Examination of the 29 infections (Table 2) reveals large variation across infections, as expected, while the average infection prevalence of the three population groups within a single infection tends to be similar (Figure 2A), consistent with the statistical analysis above. The similarity of the population groups within an infection is even more evident in the 13 most representative infections (Figure 2B). M and S molecular forms were analysed further by plotting the ratio of infection prevalence in M form *versus* S form mosquitoes across all 29 infections (Figure 3). There is no significant difference or even a trend between M and S infection prevalence in either direction, consistent with the statistical results above. It is informative to examine the distribution of all 29 infections (open circles in Figure 3) as compared to the 13 most representative infections (filled circles in Figure 3). Of the 13 infections, almost all are tightly clustered around the null average of zero difference in infection prevalence between M and S form (9/13, ~70% with differences in infection of ≤10%). However, of the 29 infections, only nine of them (31%) show an M *versus* S infection difference of ≤10%, and all of these are part of the 13-infection subset. Thus, with greater representation of both molecular forms within each of the 13 infections, the measured infection difference between molecular forms becomes smaller. The study reaches the conclusion that the greater dispersion of infection difference in the 29 infections is simply noise and not actual differences between M and S form. Consistent with this observation, the 29 infections displayed a variance of the absolute difference in prevalence between M and S forms (σ=317.2) that is 2.5 times the variance of infection difference in the 13 infections (σ= 125.7). Overall, these results indicate that much of the noise in infection distribution due to extraneous variables can be excluded by making direct comparison between population groups of adequate sample size within an infection, and then combining those results over multiple independent infections using Fisher’s method (see Methods).

**Table 2:** Sample size of individual population groups and overall infection prevalence for all 29 infections, with the subset of 13 most representative infections containing ≥seven individuals from each of the three population groups listed first in bold

**Infection**	**A. arabiensis (n)**	**A. gambiae M form (n)**	**A. gambiae S form (n)**	**Infection prevalence (%)**
**1**	**33**	**13**	**14**	**33.3**
**2**	**28**	**9**	**7**	**9.1**
**3**	**24**	**9**	**12**	**37.8**
**4**	**37**	**10**	**12**	**54.2**
**5**	**29**	**10**	**24**	**38.1**
**6**	**24**	**17**	**8**	**63.2**
**7**	**27**	**25**	**9**	**31.1**
**8**	**31**	**7**	**14**	**22.6**
**9**	**9**	**13**	**18**	**27.5**
**10**	**31**	**9**	**19**	**61.0**
**11**	**26**	**27**	**37**	**50.0**
**12**	**16**	**8**	**25**	**30.6**
**13**	**30**	**22**	**44**	**65.6**
14	16	2	6	20.8
15	16	2	11	69.0
16	47	6	22	68.0
17	31	21	2	24.1
18	12	6	1	73.7
19	12	12	1	28.0
20	19	7	1	48.1
21	45	5	7	17.5
22	56	2	9	34.3
23	65	1	8	77.0
24	26	5	2	63.6
25	11	1	7	26.3
26	27	5	14	15.2
27	23	12	3	39.5
28	11	6	26	27.9
29	20	5	43	35.3
Total for 29 infections	782	277	406	
Total for 13 most representative infections	345	179	243	

### Infection intensity among population groups

**Figure 4:**
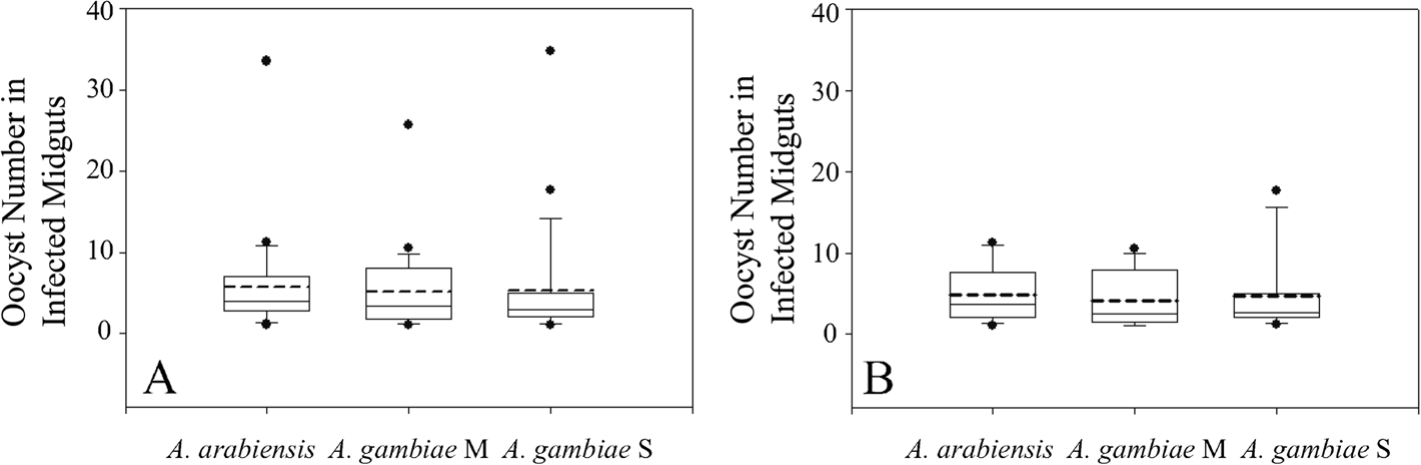
***Plasmodium falciparum *infection intensity does not differ across population groups. A**. Oocyst infection intensity of the three population groups, *A. gambiae* s.s. M form, S form and *A. arabiensis*, was calculated individually for each of 29 experimental infections, and descriptive statistics are summarized in a box plot (top and bottom of the box, 75^th^ and 25^th^ percentile; solid line within the box, median; dashed line within the box, mean; error bars, 90^th^ and tenth percentiles; dots, outliers). **B**. As in **A**, but only for the subset of 13 most representative infections. Neither the 29-infection dataset in **A** nor the 13-infection subset in **B** display a significant difference for infection intensity among population groups (see Results).

Infection intensity (average number of midgut oocysts in all mosquitoes with ≥1 oocyst) was also compared across population groups. For the 29 infections, the average oocyst intensity was 5.7 oocysts/midgut for *A. arabiensis*, and 5.2 and 5.3 oocysts for *A. gambiae* M and S forms, respectively (Figure 4A). Pair-wise comparisons across the 29 infections showed no significant difference in infection intensity between population groups (Wilcoxon Signed Rank Test, *A. arabiensis vs A. gambiae* M form, *p*=0.323, *A. arabiensis vs A. gambiae* S form, *p*=0.144 and *A. gambiae* M form *vs A. gambiae* S form *p*=0.958). For the subset of 13 infections, the results are similar (Figure 4B, *A. arabiensis vs A. gambiae* M form *p*=0.206, *A. arabiensis vs A. gambiae* S form *p*= 0.414 and *A. gambiae* M *vs A. gambiae* S form *p*=0.893). As with infection prevalence, there is more variation in infection intensity across independent infections, and much less variation within infections (Figure 2, see points).

**Figure 5:**
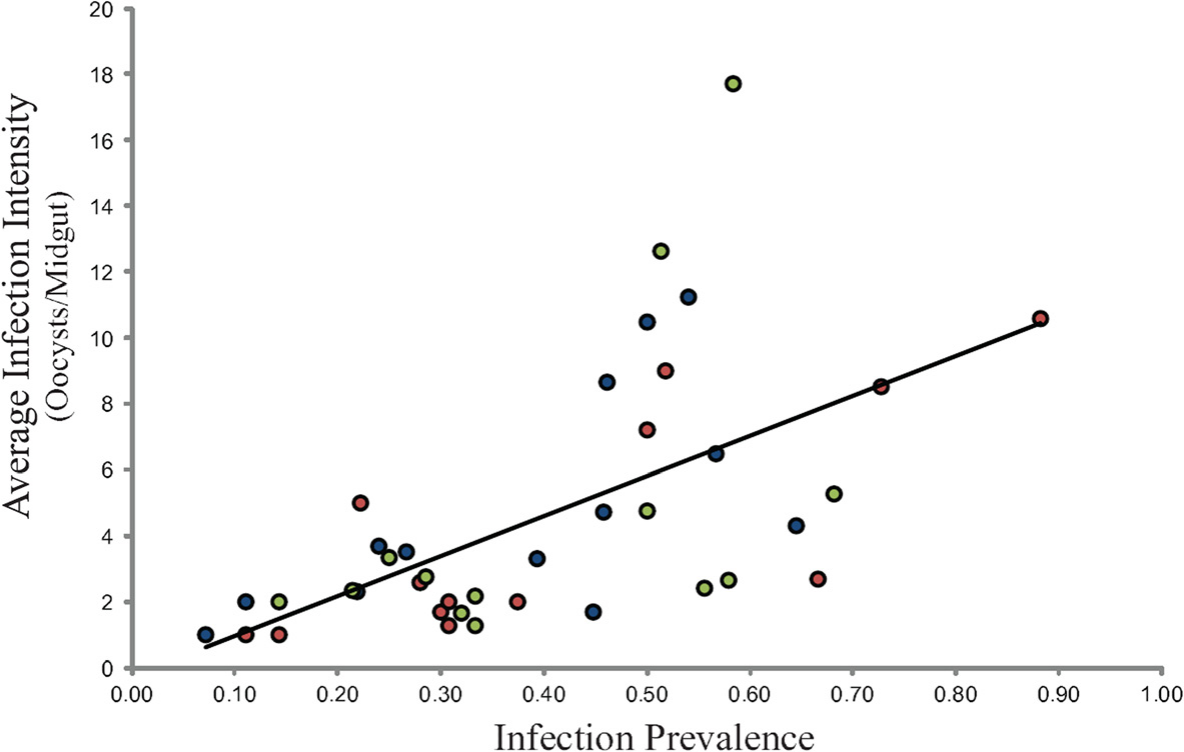
***Plasmodium falciparum *oocyst intensity correlates with infection prevalence in all population groups of *Anopheles gambiae *s.l.** Data are *A. arabiensis* (blue circles), *A. gambiae* M form (red circles) and *A. gambiae* S form (green circles). Plotted line indicates significant correlation of infection prevalence with oocyst intensity (*p*<0.001, r^2^=0.36).

In addition, the correlation between infection prevalence and infection intensity in this unique large sample set of mosquito infection phenotypes was examined (Figure 5). There is a significant trend for increased average oocyst intensity with increased infection prevalence (*p*<0.001, r^2^=0.36), and this effect is also the same for all population groups individually (*A. arabiensis p*=0.020, *A. gambiae* M form *p*=0.002, *A. gambiae* S form *p*=0.085).

## Discussion

In this study using a large sample set of mosquitoes from 29 independent infections across four malaria transmission seasons, there is no difference between the M and S molecular forms of *A. gambiae* in their genetic susceptibility to infection by *P. falciparum*, as measured by oocyst prevalence or intensity after exposure to blood meals from natural gametocyte carriers in Burkina Faso. Further, there is no difference in infection susceptibility between *A. gambiae* s.s. and its sister species, *A. arabiensis*. These findings are in accord with several previous studies that found no difference in natural infection rates between M and S form mosquitoes [23-25,44]. As mentioned above, there are multiple potential reasons why measures of genetic susceptibility presented here, and natural infection rates presented in the cited work, might differ. Natural infection rates do not control for, and in fact summarize, additional factors beyond genetic susceptibility, such as differential human feeding rates, age at infection, longevity, and exposure to other pathogens that could influence immune status. The natural infection studies and this study of genetic susceptibility, taken together, query the effects of genetics, ecology and behaviour on vectorial capacity of the M and S molecular forms. The observation that the outcome does not differ between studies done in entirely different ways would appear to firmly reject the hypothesis of differences in infection or vectorial capacity of the M and S molecular forms, at multiple geographic locations in West and Central Africa. Thus, it is concluded that the M and S molecular forms, along with *A. arabiensis*, are all equally dangerous vectors of human malaria.

There are two published accounts that report a difference in genetic susceptibility between M and S forms, and are thus at odds with the current results and the previous wild collections. In one of the reports, the mosquitoes tested were from pure M or S form laboratory colonies [26]. In that study, the S form colony was found to be more susceptible than the M form colony. However, colonization of mosquitoes is associated with strong selection pressure and genetic bottlenecks due to the founder effect [45]. Studies of colonization and domestication in other organisms also reveal large random and non-random loss of genetic diversity [46]. Thus, phenotype results derived from colonies pertain only to those specific colonies, and cannot serve as a model for the source population without adequate replication using multiple independent colonies of each source population or subgroup.

The other discrepant study found the opposite direction of difference, that is, higher susceptibility of M form mosquitoes [27]. There are multiple differences between that study and the current one, including their use of RT-PCR for parasite detection, while here microscopy was used. There may also be differences in larval collection methods, since here small numbers of larvae were collected per site over a large number of sites, to avoid sibling bias due to genetically related mosquitoes, and any potential influence of larval site microbial flora is also controlled for (see Methods), in order to specifically query mosquito genetics. In addition, in the current study, tested mosquitoes were collected at multiple times of the season across four years to control for temporal bias. These methodological details are not described in Boissière *et al.*, so it is not possible to evaluate if they could underlie the different results. Most importantly, the current study demonstrates that the infections that contained the largest representation of both molecular forms in the same infection displayed the least infection difference between molecular forms, while the infections that were less representative of both forms were the most likely to capture uncontrolled experimental noise and consequently deviate artifactually from the null average. Boissière *et al.* generated 18 infections from a sympatric zone that may have included both molecular forms in the same infection, but the sizes of infection samples or their relative M and S form composition was not reported. Consequently, it is not possible to identify potential sources of the different results between that study and the current report.

The results presented here indicate that an important requirement for comparative susceptibility studies is the inclusion of all groups being compared in the same parasite challenge, and at the largest feasible sample size per infection. Based on the principles of statistical sampling, comparisons involving the largest and most representative infection samples should detect the most robust, statistically significant differences in infection prevalence or intensity, if they exist. Conversely, even in the absence of a true biological difference in susceptibility between population groups, smaller and less representative infection samples, which suffer the most stochastic variation due to experimental noise, will still display the largest (but artifactual) differences in infection.

It cannot be ruled out that M and S forms in different geographic locations or ecological situations could display different infection results than those reported here. Different populations are under distinct selective pressures, including exposure to other pathogens, and this could yield local differences between M and S forms for malaria susceptibility. Also, the evolutionary and demographic history of the M and S forms is not yet clear, and complex population admixtures are observed [12,17], as well as reproductively isolated founder populations with distinct ecological characteristics such as Goundry [19], Forest-M and Mopti-M forms [5]. Thus, different populations of the M or S form might not all share the same evolutionary history, and could therefore display different response to malaria parasites. The Goundry form from Burkina Faso is significantly more genetically susceptible to *P. falciparum* than are the sympatric M and S forms combined [19], and some chromosomal forms of *A. gambiae* s.s. in Mali appear to display different levels of natural infection [23].

Populations of *A. gambiae* s.s. in at least Mali [20,21] and Burkina Faso at the current study site [22] segregate for genetic variation at immune genes between the molecular forms. For two gene loci, *TEP1* and *APL1*, signatures of positive selection and low levels of nucleotide variation are found within the M form, whereas sympatric S form mosquitoes do not display evidence of positive selection. However, despite the evidence of different evolutionary pressure on immune genes among molecular forms, the preponderance of reports including this one clearly demonstrates that the molecular genetic variation at the *TEP1* and *APL1* loci is not associated with any consistent molecular form susceptibility difference to malaria parasites in nature. It is nevertheless puzzling that the genetic differentiation between molecular forms at two genes that protect mosquitoes against *P. falciparum* in gene silencing experiments, *TEP1* and *APL1A*[21,47], is not reflected in a phenotypic difference between molecular form susceptibility in nature. Resolving this apparent paradox will require further work.

*Anopheles arabiensis* is often regarded as more exophilic and zoophilic than *A. gambiae* s.s. [48]. However, these results indicate that when *A. arabiensis* is offered the same infectious human blood meal as *A. gambiae* s.s., there is no difference in the establishment and extent of *P. falciparum* infection. This equivalent genetic susceptibility of the two vectors is important in the context of reports of that *A. arabiensis* has replaced *A. gambiae* after vector control interventions [2,3]. Even though *A. arabiensis* may not be the primary vector in many locations, it has a genetic susceptibility equivalent to that of *A. gambiae* s.s.

## Conclusions

This field-based study of natural mosquito and parasite genotypes, tested in a large number of independent experimental infections, clearly shows that there is no difference in genetic susceptibility to *P. falciparum* infection between the M and S molecular forms of *A. gambiae*. It also establishes that *A. arabiensis* is just as likely to become infected with *P. falciparum* as *A. gambiae* s.s. when exposed to an infective blood meal.

## Competing interests

The authors declare that they have no competing interests.

## Authors' contributions

AG, WMG, KE, MMR, N’FS and KDV conceived and designed the experiments. AG, AS, AT and ZS performed the experiments. AG, WMG, KE, MMR, GBK, N’FS and KDV analysed the data and wrote the manuscript. All authors read and approved the final manuscript.
